# Nuclear resonant scattering from ^193^Ir as a probe of the electronic and magnetic properties of iridates

**DOI:** 10.1038/s41598-019-41130-3

**Published:** 2019-03-25

**Authors:** Pavel Alexeev, Olaf Leupold, Ilya Sergueev, Marcus Herlitschke, Desmond F. McMorrow, Robin S. Perry, Emily C. Hunter, Ralf Röhlsberger, Hans-Christian Wille

**Affiliations:** 1 Deutsches Elektronen-Synchrotron DESY, Notkestraße 85, 22607 Hamburg, Germany; 2 The Hamburg Centre for Ultrafast Imaging, Luruper Chaussee 149, 22761 Hamburg, Germany; 3 London Centre for Nanotechnology and Department of Physics and Astronomy, University College London, Gower Street, London, WC1E 6BT United Kingdom

## Abstract

The high brilliance of modern synchrotron radiation sources facilitates experiments with high-energy x-rays across a range of disciplines, including the study of the electronic and magnetic correlations using elastic and inelastic scattering techniques. Here we report on Nuclear Resonance Scattering at the 73 keV nuclear level in ^193^Ir. The transitions between the hyperfine split levels show an untypically high E2/M1 multi-polarity mixing ratio combined with an increased sensitivity to certain changes in the hyperfine field direction compared to non-mixing transitions. The method opens a new way for probing local magnetic and electronic properties of correlated materials containing iridium and provides novel insights into anisotropic magnetism in iridates. In particular, unexpected out-of-plane components of magnetic hyperfine fields and non-zero electric field gradients in Sr_2_IrO_4_ have been detected and attributed to the strong spin-orbit interaction in this iridate. Due to the high, 62% natural abundance of the ^193^Ir isotope, no isotopic enrichment of the samples is required, qualifying the method for a broad range of applications.

## Introduction

There is burgeoning interest in understanding the physical properties of systems which are simultaneously subject to strong spin-orbit coupling (SOC) and electron correlations, as exemplified by recent studies of novel electronic and magnetic phases displayed by various 4d and 5d transition metal oxides (TMOs)^1,2^.

At one level, SOC introduces another competing energy scale, producing unexpected electronic states. This is the case for the so-called spin-orbit Mott insulator in iridate perovksites which would otherwise be expected to be metallic in the absence of SOC^3,4^. At another, more profound level, the SOC fully entangles spin and orbital degrees of freedom such that the magnetic interactions acquire an anisotropic, bond-directional nature – the Kitaev interaction – which can compete with the conventional isotropic Heisenberg term dominating 3d systems^5^. The resulting Kitaev-Heisenberg model is proving to be extremely rich displaying a plethora of topological quantum phases including spin-liquids, superconductivity, etc., the exploration of which is in its infancy^6–8^. Further impetus for studying 4d and 5d TMOs stems from the rich possibilities offered by nano-structuring these materials, finding potential applications as biosensors, spintronic devices, catalysts, etc.^9–12^.

The iridate perovskites forming the Ruddlesden-Popper series of compounds Sr_*n*+1_Ir_*n*_O_3*n*+1_ play a central role in the evolution of the field of systems combining SOC and electron correlations^13^. Sr_2_IrO_4_ (n = 1) was the first example of the new class of spin orbit Mott insulators^4^ which has attracted considerable interest due to the similarities of its magnetism, and to a certain extent its electronic structure, to La_2_CuO_4_, the parent compound of high-temperature superconductors^14–18^. Indeed, potassium doped onto the surface of Sr_2_IrO_4_ has been shown to induce a d-wave gap similar to that displayed by superconducting cuprates, although definitive proof of superconductivity in the iridate perovskites has not yet been produced^19,20^. Sr_3_Ir_2_O_7_ (n = 2) is a marginal spin-orbit Mott insulator, in the sense that it can be transformed to unusual confined metallic phase (conducting in the a-b plane only) for pressures above 55 GPa, although the details of key properties such as the magnetism of the high-pressure phase are unknown^21–23^.

Revealing the nature of the electronic and magnetic correlations in iridates presents certain challenges which need to be overcome. These include the fact that the physics depends on a hierarchy of competing energy scales, requiring the characterisation of electronic and magnetic correlations over large ranges of energy and length scales. Second, single crystals of novel materials are often initially very small (in some cases no larger than 10 *μ*m), meaning that methods with high sensitivity have to be developed. X-ray resonant scattering, both elastic (REXS)^16,24^ and inelastic (RIXS)^15,25,26^, from the Ir 5d electrons has proven to be especially useful, particularly so as neutron techniques are more challenging due to the low sensitivity of the technique and the high neutron absorption cross section of Ir^27,28^.

In this report we establish Nuclear Resonance Scattering (NRS) on ^193^Ir at 73 keV as a complementary probe to REXS and RIXS for probing the electronic properties and magnetism of iridates. The main advantages of NRS are its exquisite sensitivity to the magnitude and direction of the electric and magnetic hyperfine fields, rendering it uniquely capable of revealing subtle changes to crystallographic and magnetic structures^29–31^. Moreover, the high photon energy of the ^193^Ir resonance^32^ opens the possibility of studying iridates under extreme conditions of pressure, such as the insulator to metal transition displayed by Sr_3_Ir_2_O_7_. In general, the high energy (*E*) X-ray regime (70 ≤ *E* ≤ 100 keV) is challenging for NRS experiments as the design of an efficient monochromator is constrained by the angular acceptance of Bragg reflections, which decreases fast, proportional to 1/*E*^2^. However, the low angular divergence of modern synchrotron sources up to high photon energies now allows such experiments to be performed effectively.

Conventional Mössbauer spectroscopy on Ir has been performed several decades ago^32,33^, but did not become widespread, because the preparation of radioactive sources was notoriously difficult. NRS, on the other hand, does not require a radioactive source. Moreover, the narrow collimation, small beam size and high flux accessible at modern synchrotron radiation sources favor NRS studies of nanostructures^34,35^ and small samples at extremely high pressures and temperatures^29,30,36^. Natural Ir occurs in two stable isotopes, ^191^Ir and ^193^Ir. The 73 keV transition in ^193^Ir with nuclear spins of 3/2 and 1/2 of ground and excited state, respectively, is most favorable for NRS studies due to the high, 62% natural abundance of the ^193^Ir isotope and a comparatively long natural lifetime of 8 ns.

## Results and Discussion

**Figure 1:**
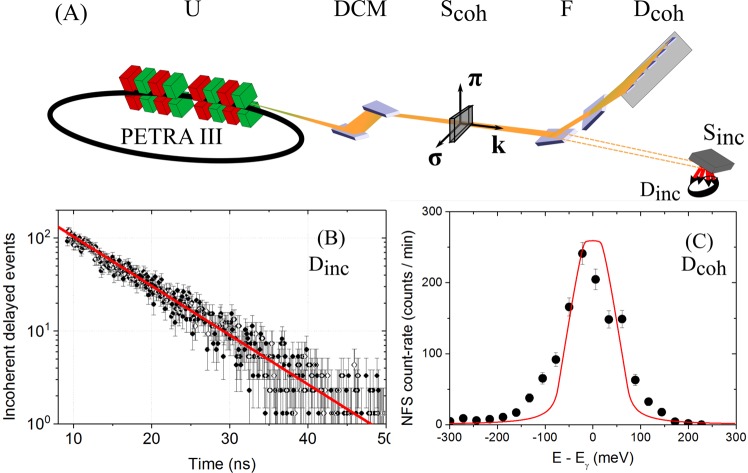
(**A**) Experimental setup with U - undulator source, DCM - double crystal monochromator, F - filtering optics, D_coh_ and D_inc_ - nuclear forward and nuclear fluorescence APD detector, respectively, and S_coh_ and S_inc_ - samples for forward and incoherent scattering experiment, respectively. (**B**) Time spectrum of delayed nuclear fluorescence (black dots) and exponential decay with time constant *τ*_0_ = 8.4(2) ns (red line). (**C**) Spectrum of coherent delayed events. The red line shows the instrumental function predicted by the dynamical theory of x-ray diffraction.

The experiments were conducted at the Dynamics Beamline P01 at PETRA III (DESY, Hamburg)^37^. The storage ring was operated in 40-bunch top-up mode providing a stable 100 mA ring current. The experimental setup (Fig. 1(A)) included a nitrogen-cooled double-crystal Si(311) monochromator which reduced the energy bandwidth of the 73 keV photons to about 8(1) eV. In order to reduce the detector load, the energy bandwidth around the nuclear resonance of photons transmitted by the sample was filtered via Bragg reflections from two specially designed Si crystals (F, Fig. 1(A)). The first crystal with an asymmetric (440) reflection collimates the beam for matching the acceptance of the subsequent (642) reflection which reduces the energy bandwidth to about 150 meV. Tuning of the photon energy to that of the nuclear resonance and measurement of the lifetime of the excited state was performed by monitoring delayed nuclear fluorescence from an Ir metal foil by a large area avalanche photo diode (APD) detector (D_inc_, Fig. 1(A)). The nuclear forward scattering (NFS) was detected by a fast detector array consisting of 16 APDs (D_coh_, Fig. 1(A))^38,39^. The fast detector array and very high bunch purity in the PETRA storage ring enabled the counting of delayed photons as early as 3 ns after the excitation pulse with a time resolution of about 0.6 ns.

The nuclear resonance was found 3.211 keV below the K- edge of Ir at 76.111 keV, and its energy was determined to be 72.90(8) keV. This value is in good agreement with the frequently reported literature value of 73.0(5) keV^40^, though it is lower than the more precise value 73.045(5) keV obtained in ref.^41^ from the measurement of internal conversion. The reason for the latter is unclear as in both measurements the maximum of the derivative of the edge absorption curve was used as a reference. Furthermore, the reference value of the edge used in ref.^41^ is 76.101 keV (10 eV lower compared to the recently defined value we used), which increases the disagreement. Fitting the time spectrum of delayed nuclear fluorescence with an exponential decay function (Fig. 1(B)), we determined the natural lifetime to be 8.4(2) ns, in accordance with the lifetime value of 8.8(2) ns reported in ref.^40^ and slightly lower than the one reported in ref.^41^ of 8.78 ns (no error given). The corresponding resonance linewidth is 78(2) neV. Using the NFS setup, we measured an instrumental function of the filtering optics (Fig. 1(C)). Its width of 158(8) meV (FWHM) is close to that of 112 meV (FWHM) predicted by dynamical theory (Fig. 1(C), red line). The broadening can be related to the imperfections in the bulk silicon utilized for the crystals.

**Figure 2:**
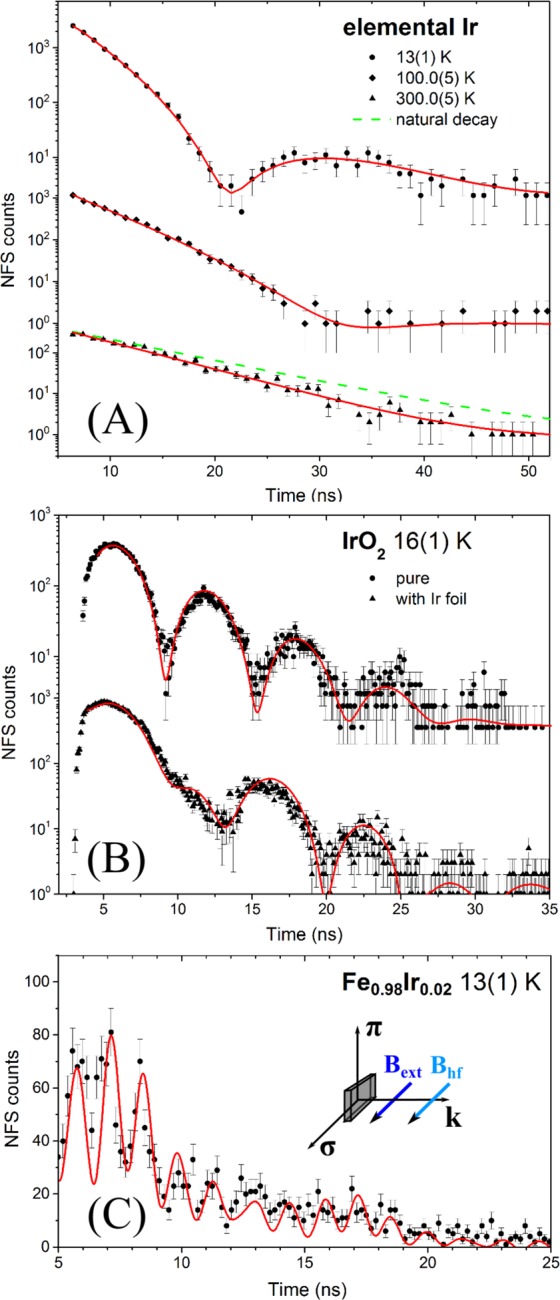
NFS time spectra of: (**A**) Ir foil, (**B**) IrO_2_ powder, (**C**) Fe_0.98_Ir_0.02_. Black markers show experimental data and the red lines show fits by nuclear dynamical scattering theory. Green dotted line in the lower graph of (**A**) shows the natural decay of the 73 keV state. For better visibility (**C**) is plotted in linear scale; the inset shows the scattering geometry, directions of external magnetic field *B*_*ext*_ and hyperfine field *B*_*hf*_.

In order to demonstrate the feasibility of the technique we performed NFS measurements on elemental Ir and on IrO_2_. Both materials have been studied earlier by conventional Mössbauer spectroscopy^42^; these are used here as references for validation of data treatment routines in the time domain. While elemental Ir shows a single resonance line, Ir in IrO_2_ exhibits an Ir^4+^ state with a pure electric hyperfine interaction^42^. NRS time spectra of 100 *μ*m thick foil of elemental Ir have been acquired in half an hour (Fig. 2(A)) at signal countrates of about 7 s^−1^. We observed a shift of beating minima to later times with increasing temperature due to the decrease of the Lamb-Mössbauer factor (Fig. 2(A), lower graphs). Since the temporal beating pattern can be fully described as dynamical beats corresponding to the small sample thickness^43^, hyperfine interactions can be ruled out, in accordance with the cubic lattice and paramagnetism of the elemental Ir^44^. Fitting the temperature dependence of the Lamb-Mössbauer factor with the Debye model^45^, we determined the Debye temperature of Ir to be 309(30) K. This value is in good agreement with the literature value of 335(13) K^46^. NFS time spectra of the IrO_2_ powder sample are shown in Fig. 2(B). Fitting of the experimental data (Fig. 2(B), upper graph) was performed with the CONUSS software^47^ extended by its author to take the high mixing ratio of the E2/M1 multipole radiation into account^42,48^. Special cases of the NRS theory for ferro-magnetic and anti-ferromagnetic arrangements considering high mixing ratios are rolled out in detail in the Supplementary Information. Where suitable, a comparison to the simple M1 case in ^57^Fe is also given there. Fitting the data yielded a quadrupole splitting ${\rm{\Delta }}{E}_{Q}=\tfrac{eQ{V}_{zz}}{2}$ of 2.76(2) mm/s (8.96(7) Γ_0_) (*e* is the elementary change, *Q* is the quadrupole moment, *V*_*zz*_ is the electric field gradient (EFG) along the quantization axis). This value is in excellent agreement with the value of 2.71(6) mm/s reported in ref.^49^. Assuming an axially symmetric EFG we obtain a value of *V*_*zz*_ = 1.71(1) · 10^18^ V/cm^2^ for the main component of the EFG which is two orders of magnitude higher than in the isostructural 4d-RuO_2_ reported in ref.^50^. The EFG in IrO_2_ is therefore mostly determined by valence 5d-electrons because of: (i) three times lower shielding of the Ir nucleus from the valence electrons than from the surrounding ions^42,51^ and (ii) more elongated 5d-orbitals in IrO_2_ providing a potentially higher EFG^45^. In order to measure the isomer shift of Ir^4+^ in IrO_2_, we introduced an Ir metal foil as a single line reference absorber and acquired a NFS time spectrum of the combined setup (Fig. 2(B), lower graph). From the evaluation of this dataset we obtained an isomer shift of −0.89(5) mm/s in IrO_2_ relative to Ir metal, which is in good agreement with the value of −0.93(1) mm/s reported in ref.^52^.

To develop the method for studies of magnetic materials, we measured NFS from the ferromagnetic alloy Fe_0.98_Ir_0.02_ in an external magnetic field of 0.53(5) T. Dilute alloys of Fe_1−*x*_Ir_*x*_ ($x\le 0.1$) show nearly pure magnetic hyperfine interactions^53,54^, and the hyperfine fields in these alloys are the highest for all known compounds with d-elements^55^. The large hyperfine fields lead to very fast oscillations in the temporal beat patterns of NFS and therefore provide the best benchmark of time resolution of the setup. The NFS time spectrum of a 1.6 mm thick sample of Fe_0.98_Ir_0.02_ exhibits extremely fast oscillations with a period of ≈1.5 ns (Fig. 2(C)). Notably, despite the before mentioned high E2/M1 mixing ratio the Fe_0.98_Ir_0.02_ NFS spectrum shows a very regular beating pattern, significant for an here almost pure, two transition line spectrum. At first glance this is surprising as even the pure M1 case (e.g. for ^57^Fe) shows a more complicated spectrum at this specific magnetic field direction. The reason for the spectrum with two transition lines is the E2/M1 mixing parameter, whose value is close to −$\sqrt{1/3}$, so that M1 and E2 transition amplitudes in the mixed M1/E2 case can cancel each other for specific transitions in ^193^Ir (see Supplementary Information). We refine the value of the hyperfine field to 133(1) T, which is in good agreement with the value of 140(2) T reported for Fe_0.973_Ir_0.027_ in ref.^32^.

Having validated the NRS technique by studying relevant reference samples, we applied it to exploring the magnetism and electronic properties of two iridates with very different properties, SrIrO_3_ and Sr_2_IrO_4_.

**Figure 3:**
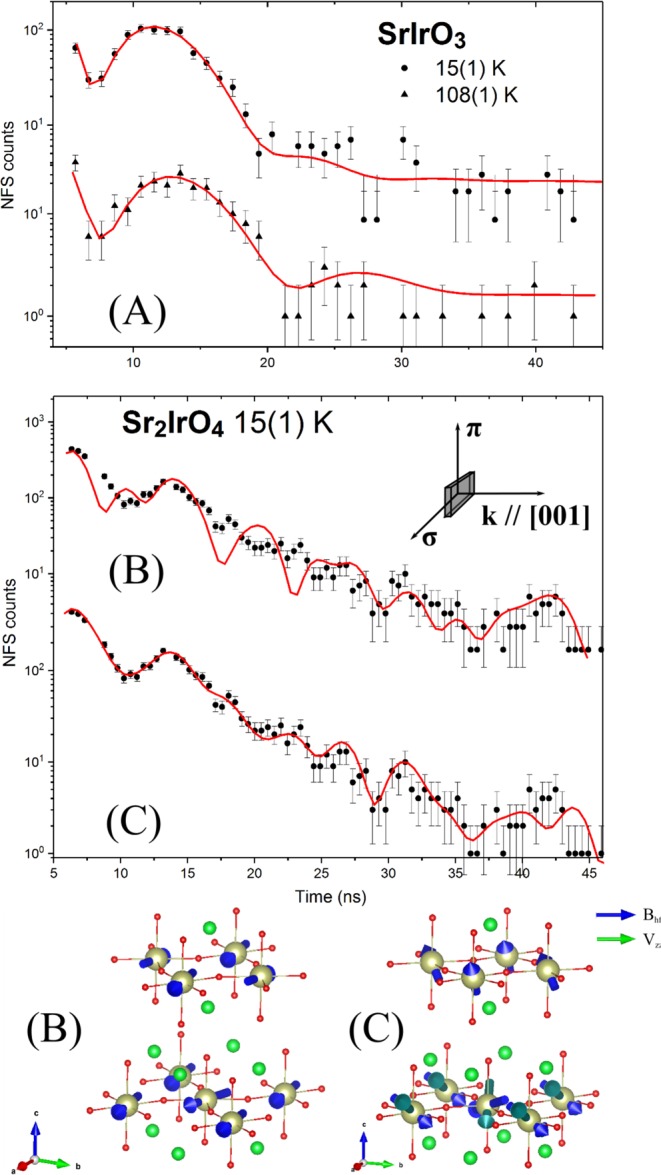
(**A**) Temperature dependent NFS time spectra of SrIrO_3_; (**B**) NFS time spectra of Sr_2_IrO_4_. The inset shows the scattering geometry. The red lines are fits by nuclear dynamical scattering theory: (**B**) assuming hyperfine fields in the basal plane and (**C**) with hyperfine planes tilted from the basal plane. The directions of the magnetic hyperfine fields *B*_*hf*_ and EFG quantization axes *V*_*zz*_ for the corresponding model fit in (**B** and **C**) are shown at the bottom.

SrIrO_3_ in its ambient pressure, monoclinic phase studied here is a low-carrier, Dirac semimetal displaying enhanced Pauli paramagnetism; properties similar to those exhibited by the high-pressure, distorted perovskite variant^13,56,57^. Due to its impact on the electronic anisotropy and EFG resulting from it, the spin-orbit interaction in SrIrO_3_ can be studied by probing the temperature dependent quadrupole splitting of the ^193^Ir nuclear levels analogous to the case^58^ of ^57^Fe. For a SrIrO_3_ powder sample we obtained a quadrupole splitting of 1.24(5) mm/s (4.0(2) Γ_0_) at 15 K (Fig. 3(A), upper graph), in very good agreement with the value of 1.26 mm/s measured at 4 K earlier^52^. Magnetic hyperfine interactions can be ruled out, in accordance with paramagnetism in this compound in the temperature range investigated^56^. The quadrupole splitting decreases with temperature and reaches a value of 1.08(5) mm/s (3.5(2) Γ_0_) at 108 K (Fig. 3(A), lower graph), which can be related to the presence of a gap in the electronic ground state. To the best of our knowledge, no change of Ir coordination symmetry is reported for the temperature range investigated. Therefore the temperature dependent change in quadrupole splitting can be exclusively attributed to the thermal population of electronic levels, supporting the evidence of semimetal-like electronic band structure^57^ in SrIrO_3_ and showing the decisive impact of the large temperature-invariant distortions in IrO_6_ octahedra onto the electronic structure in this compound.

The unique strength of the NRS technique is its high sensitivity to the magnitude and orientation of magnetic fields and electric field gradients at the local Ir sites (for details see Supplementary Information). This allowed us to gain new insights into the magnetic order of the Sr_2_IrO_4_ perovskite. The Sr_2_IrO_4_ crystals in this study have a form of platelets with lateral size of 2 × 3 mm^2^ and thickness of about 30–70 *μ*m. Five Sr_2_IrO_4_ crystals have been stacked in order to increase the NFS signal. They were aligned with the (001) plane perpendicular to the incident beam (inset Fig. 3(B,C)). The entire incident beam was accepted by the sample. Details on the sample preparation and alignment are given in the Supplementary Information.

**Figure 4:**
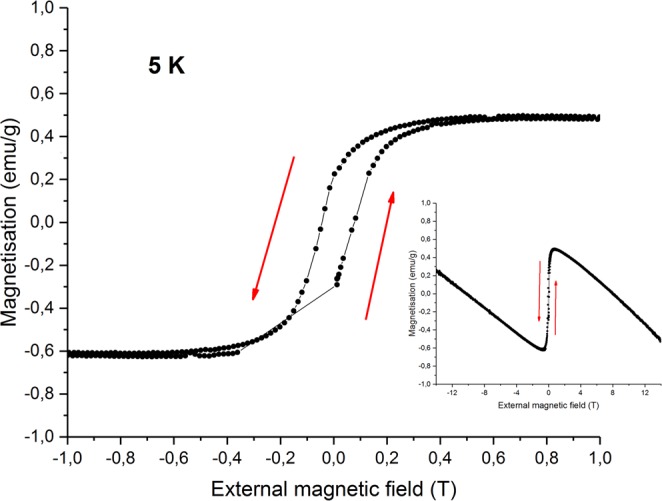
Hysteresis of magnetization of the Sr_2_IrO_4_ sample at 5 K with external field applied perpendicular to the c–axis (magnified in the −1 to 1 T range). Inset: same for the full range, from −14 to 14 T.

Energy-dispersive X-ray spectroscopy (EDX) and magnetization measurements suggest slight oxygen deficiency in the Sr_2_IrO_4_ sample. EDX indicates the chemical composition Sr_1.83(1)_IrO_3.89(2)_. Though EDX is not precise in determining oxygen content, the magnetization hysteresis of the samples is very similar to that of the oxygen deficient sample with chemical composition Sr_2.08_IrO_3.86_ reported in the ref.^59^ (Fig. 4). No abrupt changes in magnetisation were observed around 0.2 T.

The temporal beat pattern in the time spectrum of Sr_2_IrO_4_ shows both magnetic and electric hyperfine interactions (Fig. 3(B,C)). We obtained a hyperfine field of 24.2(2) T which is in a very good agreement with the value of 24 T reported by Mössbauer spectroscopy in ref.^42^. Any model with in-plane hyperfine fields fails to explain the measured time spectrum (Fig. 3(B)). Taking into account oxygen deficiency and associated distortion of tetragonal symmetry^28^, the local symmetry of Ir in Sr_2_IrO_4_ permits the existence of magnetic components along the c-axis (see Supplementary Information). Introducing a 30° tilting angle of hyperfine fields to the a-b plane into the model fit provides a very good statistical quality of the fit to the measured time spectrum (Fig. 3(C)), supporting the existence of out-of-plane components of the magnetic field at the Ir sites. One has to note that the direction of hyperfine field and magnetic moment do not need to coincide^60^. Especially, the effect is expected in the presence of a significant orbital field contribution usually observed as an anisotropy of the electronic g-factor^45,60^. A high electronegativity of Ir favors high covalency of Ir-O bonds, reducing the Fermi contact field and increasing the orbital field contribution to the hyperfine field^61–63^. A strong anisotropy in the electronic *g*-factors in Sr_2_IrO_4_ observed by ESR in ref.^64^ supports this hypothesis. Considering the electric field at the Ir nuclei in Sr_2_IrO_4_, we observe an axially symmetric EFG with a magnitude of 1.1(1) · 10^18^ V/cm^2^ in the [001] direction. The presence of an EFG is reasonable in view of distortion of the IrO_6_ octahedra^3^, oxygen deficiency, and the evidence of a non-zero EFG in the isostructural Sr_2_RuO_4_^65^. The non-zero EFG in Sr_2_IrO_4_ and the out-of-plane components of the magnetic hyperfine field found here might be attributed to the non-zero angular momentum of the outer electrons, arising from the reduced symmetry of the IrO_6_ octahedra; temperature dependent measurements can provide further information on the origin of this phenomenon.

## Conclusion

In conclusion, we have established Nuclear Resonance Scattering at the 72.90(8) keV level in ^193^Ir as a new synchrotron-based technique for the studies of magnetism and electronic properties of iridates. A huge 133(1) T hyperfine field in dilute Fe_0.98_Ir_0.02_ alloy has been detected via NRS. Moreover, we found a thermally induced decrease of the electric field gradient across the Ir nuclei in SrIrO_3_ and observed a non-zero EFG and tilting of hyperfine fields from the basal plane in Sr_2_IrO_4_ that should stimulate further investigations to relate structural and electronic properties in the iridates. All samples contained ^193^Ir in its natural abundance; no preparation of radioactive sources is required and no line broadening due to the source is present. NRS at ^193^Ir is sensitive to dilute systems and spin structures, providing a valuable input for studies to relate magnetism and spin-orbit interactions in iridates, e.g. in strong magnetic fields^24,33^, or under confinement in nanomaterials^9,11,12^ and heterostructures^66^. The oxidation state of iridium and crystal fields at Ir ions can be tracked via measurements of isomer shift and quadrupole interactions at the Ir nucleus, respectively.

## Supplementary information

Supplementary Information to Nuclear resonant scattering from $^{193}$Ir as a probe of the electronic and magnetic properties of iridates
