# Supplementary material for: Nuclear resonant scattering from 193Ir as a probe of the electronic and magnetic properties of iridates
Source: Sci Rep. 2019 Mar 25;9:5097. doi: 10.1038/s41598-019-41130-3 (PMC6433947; doi:10.1038/s41598-019-41130-3)
Supplement: Supplementary file 1 — Supplementary Information to Nuclear resonant scattering from $^{193}$Ir as a probe of the electronic and magnetic properties of iridates [file 41598_2019_41130_MOESM1_ESM.pdf]

Supplementary Information to Nuclear resonant scattering from ^{193}Ir as a probe of the electronic and magnetic properties of iridates

Pavel Alexeev[1,2], Olaf Leupold[1], Ilya Sergueev[1,], Marcus Herlitschke[1,], Desmond F. McMorrow[3,], Robin S. Perry[3,], Emily C. Hunter[3,], Ralf Röhlsberger[1,], and Hans-Christian Wille[1*,]

[1]Deutsches Elektronen-Synchrotron DESY, Notkestraße 85, 22607 Hamburg, Germany
[2]The Hamburg Centre for Ultrafast Imaging, Luruper Chaussee 149, 22761 Hamburg, Germany
[3]London Centre for Nanotechnology and Department of Physics and Astronomy, University College London, Gower Street, London WC1E 6BT, United Kingdom
*hans.christian.wille@desy.de

ABSTRACT

Supplementary information about methods and basic principles used for Nuclear resonant scattering from ^{193}Ir as a probe of the electronic and magnetic properties of iridates

Sensitivity of NRS to the Direction of Magnetic Hyperfine Fields in Iridates

Nuclear multipole transitions are excited selectively, depending on the direction of the γ radiation with respect to the quantization axis defined by the hyperfine fields, and on the change Δm of the magnetic quantum numbers of the hyperfine levels involved in the transition. Synchrotron radiation is polarized, in the standard notation it's called σ polarization. This gives additional information in NFS since γ radiation from nuclear transitions is polarized, as well, rendering NFS sensitive to the orientation of hyperfine fields[1]. For instance, if B_{hf} is parallel to the wave vector $\vec{k}$, the eigenpolarizations of the nuclear transitions are left and right circular, resp., and a two line beating pattern with reduced quantum beat contrast is observed, see Fig. S1 first row, in this case M1/E2 and pure M1 radiation show the same behaviour.

For magnetic hyperfine fields in the plane orthogonal to the beam direction the linear eigenpolarizations are σ and π. When the hyperfine field is aligned parallel to the σ-polarization of synchrotron radiation, in case of pure M1 transitions, only the $\Delta m = \pm 1$ transitions are excited, which results in a 4 line beating pattern[1]. For the mixed M1/E2 transitions of ^{193}Ir one gets an unexpected 2 line beating pattern, see Fig. S1, 2nd row. This is due to the E2/M1 mixing parameter, which has a value of -0.577[2] for ^{193}Ir. This value is "accidentally" close to $-\sqrt{1/3}$ which leads to a cancelling of specific M1 and E2 transition amplitudes.

In case of a hyperfine field aligned perpendicular to the σ-polarization and the $\vec{k}$ vector of synchrotron radiation, there is no such cancelling of transitions and the mixed M1/E2 transitions exhibit a 4 line beating pattern, see Fig. S1, 3rd row.

Fig. S2 shows NFS energy and time spectra for different orientations of the magnetic hyperfine field relative to the wavevector and σ polarization of the exciting radiation for antiferromagnetic ordering. As compared to the pure M1 transition, where alignment of B_{hf} parallel to $\vec{k}$ and parallel to the σ polarization yield the same time patterns (Ref.[1] and Fig. S3), for mixed M1/E2 transitions these two field geometries can be distinguished. This benefit from the mixed M1/E2 transition is shown in Fig. S3 in more detail. There is an angular dependence on the hyperfine field direction, if in antiferromagnetic spin arrangement the magnetization is rotated in the $k-\sigma$ plane, Fig. S3 middle column. In the pure M1 case, shown for the ^{57}Fe resonance in the right column, the different orientations exhibit exactly the same quantum beat pattern. Hence, one essential feature of NFS at the 73 keV resonance in ^{193}Ir is the pronounced sensitivity to the tilt of the hyperfine fields from the basal plane (plane determined by σ- and π-polarization of the synchrotron radiation in the experimental setup).

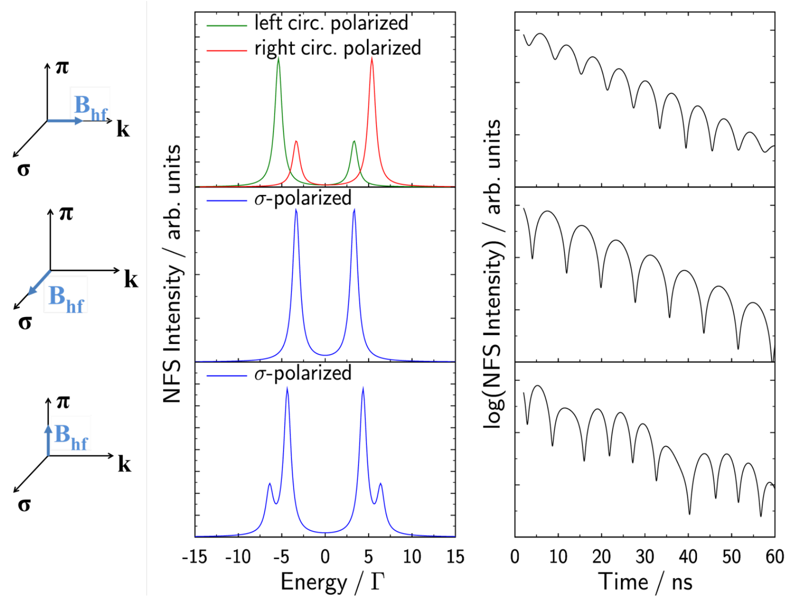

Figure S1. Energy and time spectra of NFS at the 73 keV resonance in ^{193}Ir - dependence on the direction of magnetic hyperfine field. B_{hf} (left column) depicts three selected directions of the magnetic hyperfine field relative to the incident wave with wavevector k and linear polarizations σ and π.

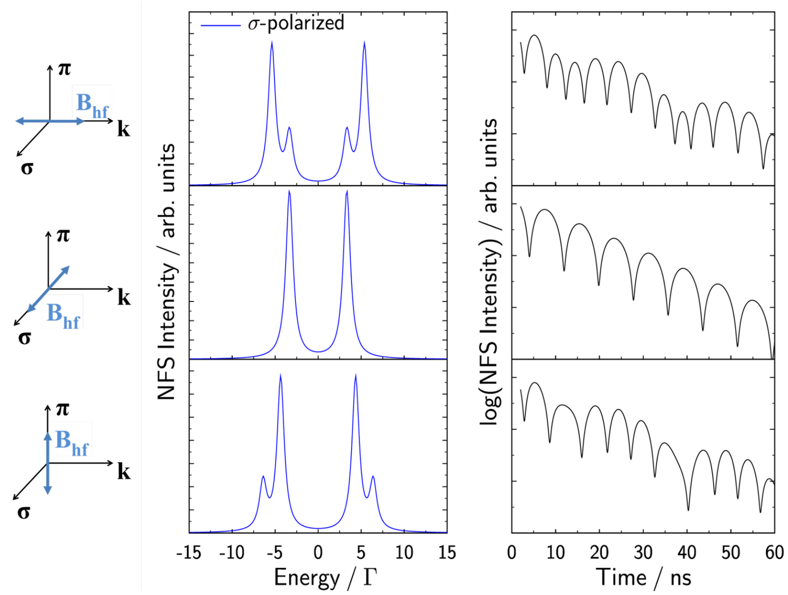

Figure S2. Energy and time spectra of NFS at the 73 keV resonance in ^{193}Ir - dependence on the direction of magnetic hyperfine fields in case of antiferromagnetic ordering. B_{hf} (left column) depicts three selected directions of magnetic hyperfine fields relative to the incident wave with wavevector k and linear polarizations σ and π.

Design of the Two-Crystal Silicon X-ray Filter

In order to prevent the detector from overloading the photon intensity has to be reduced to a certain level. This is achieved by decreasing the bandwidth around the nuclear resonant photon energy using a medium resolution monochromator or x-ray filter. The design of the x-ray filter is similar to that implemented for the NRS studies at the 67 keV resonance in ^{61}Ni[3]. The device includes two Si crystals with asymmetric Bragg reflections (Table1 and Fig. S4). A tight fixation does induce a curvature of the crystals, the effect is significant even for thick crystals, as mentioned in Ref.[3]. In the present work the crystals were placed onto the holders, without squeezing, thus, the mounting prevented curvature of the crystals.

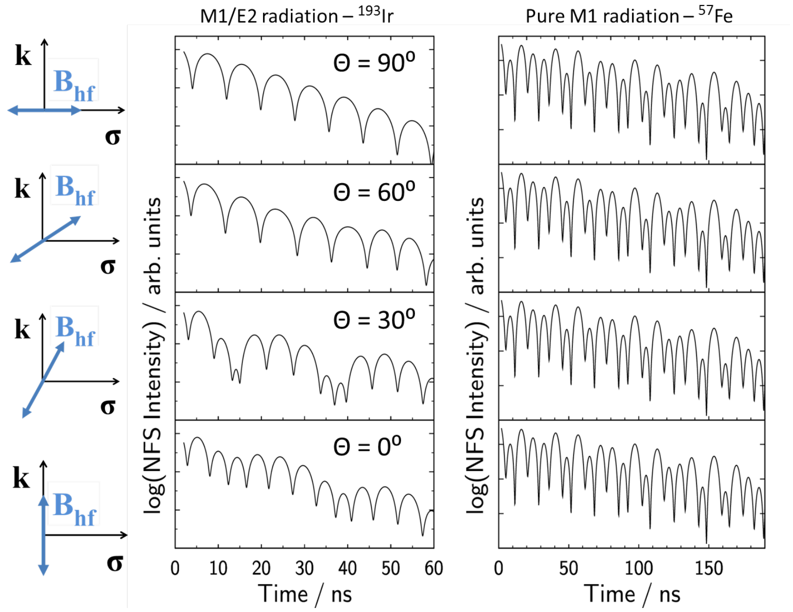

Figure S3. Comparison of NRS time spectra for mixed M1/E2 radiation (^{193}Ir) and pure M1 radiation (^{57}Fe) - dependence on the direction of antiferromagnetically ordered magnetic hyperfine fields in the $\vec{k} - \sigma$ plane. Θ is the angle between B_{hf} and the wavevector k. B_{hf} (left column) depicts the four selected directions of magnetic hyperfine fields relative to the incident wave with wavevector k and linear polarization σ.

	**First crystal**	**Second crystal**
Reflection	(4 4 0)	(6 4 2)
Bragg angle, [*degree*]	5.072	6.716
Asymmetry parameter *b*	0.11	2.6
Incident angle, [*degree*]	1.002	9.716
Angular acceptance of the incident beam, [μrad]	2.35	0.25
Angular divergence of the diffracted beam, [μrad]	0.25	0.65

Table 1. Main design parameters of the x-ray filter.

Fast APD Detector Array

The coherent NRS has been detected by a multi-element array detector built by ATIM Radiocommunications (France)[4] with 16 fast, 30 μm thin APDs S5344 from Hamamatsu Photonics (Fig. S5). For an overview on APD detectors see e.g.[5]. The diameter of each APD was 3 mm and the whole beam was accepted by the detector. The single APDs have been stacked and inclined to an angle of about 3° relative to the incident beam in order to increase detection efficiency for 73 keV photons to about 9%.

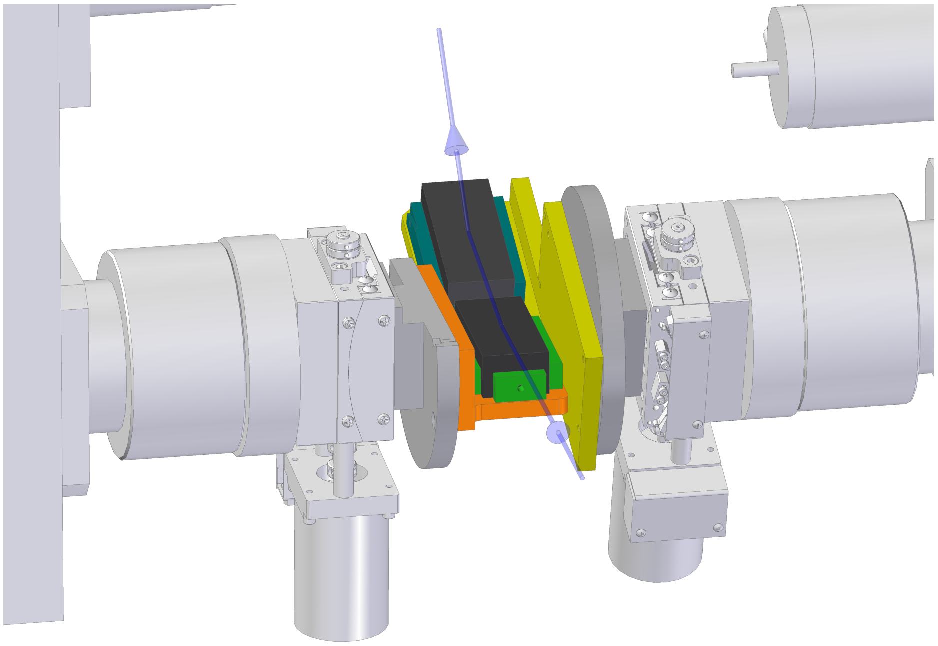

Figure S4. CAD-model of the x-ray filter setup. The blue arrows indicate the beam path.

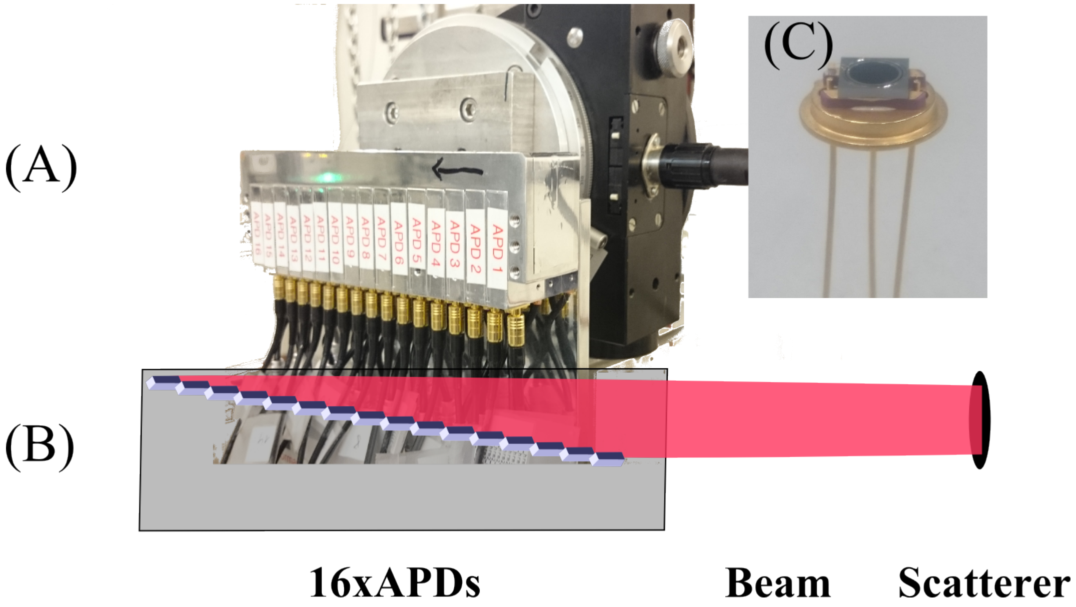

Figure S5. Multi-element APD detector: (A) Photo of the detector; (B) Principle of operation: the scattered beam is fanned out on to the 16 thin, small-area APDs inclined to the beam, providing large beam path along each APD and therefore high efficiency and time resolution; (C) Photo of single APD Hamamatsu S5344 utilized for the detector array.

Sr_2IrO_4 single crystalline sample

Sr_2IrO_4 crystals were grown using a standard high-temperature flux method[6] in a platinum crucible (volume 50 cm^3) with a platinum lid. Strontium (II) chloride (Alfa Aesar 99.9%) flux was used along with iridium (IV) oxide (99.9% Alfa Aesar) and strontium (II) carbonate (99.99% Sigma Aldrich; dried at 550°C) as starting materials. The initial constituent ratios $SrCl_2$:$SrCO_3$:IrO_2 were 7.5:1.8:1.0. The powders were ground in an agate mortar, placed into the crucible and heat cycled in a standard box furnace. The heating cycle consisted of 12 hours at 1250°C followed by a slow cool to 1100°C in 20 hours. The crucible was cooled inside the furnace to near room temperature in around 6 hours before being removed from the furnace. The crystals were removed from the matrix by sonication in warm water and were further cleaned in ethanol.

All crystals exhibit the form of platelets with (0 0 1) planes being parallel to the largest surface areas. The thickness of the crystals was about 30-70 μm and the lateral size was about 2x3 mm^2.

The orientation of the crystals was carried out using Raman spectroscopy with a 532 nm laser. Particularly, the Raman signal intensity from the B_{2g} mode (380 cm^{-1}) was measured owing to that it is maximal if the polarization of the incident laser beam is parallel to the [1 1 0] direction in Sr_2IrO_4 (Fig. S6). Assembling the sample stack under the microscope, each crystal was carefully pushed by tweezers along optical axis and the change of the focus distance was measured. Knowing the length of each crystal and change of focus depth, the deviation angle from ideally parallel crystal stacking was estimated to be about 2 to 8°. The crystals have been stacked along the beam so that the (0 0 1) plane in Sr_2IrO_4 was perpendicular to the incident beam

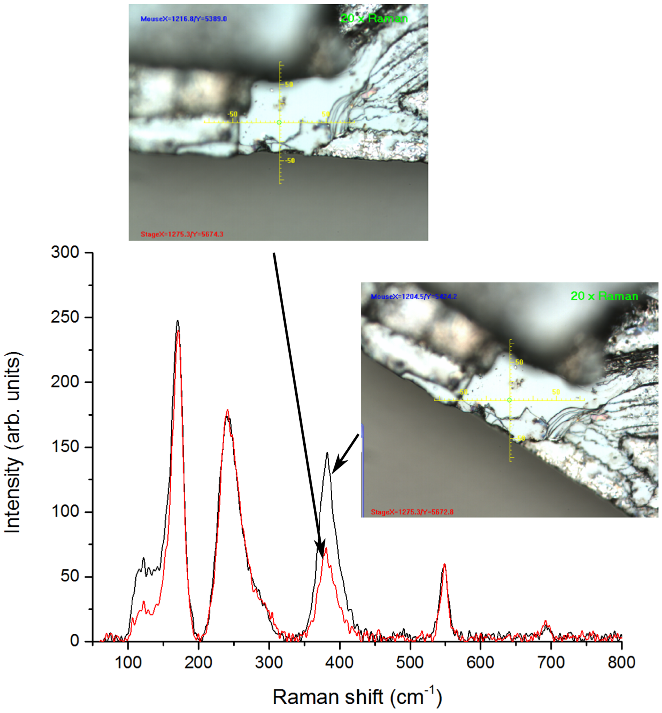

Figure S6. Alignment of the Sr_2IrO_4 crystals by Raman spectroscopy. The Raman signal from the B_{2g} mode (380 cm^{-1}) is maximal when the polarization of the incident laser beam is parallel to [1 1 0].

and the crystallographic direction [1 0 0] or [0 1 0] was parallel to the σ-polarization of the incident beam (see inset Fig. 3, B)[7].

References

1. Röhlsberger, R. *Nuclear condensed matter physics with synchrotron radiation: basic principles, methodology and applications*, vol. 208 of *Springer Tracts in Modern Physics* (Springer, Heidelberg, 2004). URL http://www.springer.com/de/book/9783540232445.

2. Wagner, F. E. Mössbauer spectroscopy with ^{191,193}Ir. *Hyperfine Interact.* **13**, 149–173 (1983). URL http://dx.doi.org/10.1007/BF01027249. DOI 10.1007/BF01027249.

3. Sergueev, I. *et al.* Nuclear forward scattering for high energy Mössbauer transitions. *Phys. Rev. Lett.* **99**, 097601 (2007). URL http://link.aps.org/doi/10.1103/PhysRevLett.99.097601. DOI 10.1103/PhysRevLett.99.097601.

4. Atim radiocommunications. https://www.atim.com/en/. Accessed: 2019-01-09.

5. Baron, A. Q. R., Kishimoto, S., Morse, J. & Rigal, J.-M. Silicon avalanche photodiodes for direct detection of X-rays. *J. Synchrotron Radiat.* **13**, 131–142 (2006). URL https://doi.org/10.1107/S090904950503431X. DOI 10.1107/S090904950503431X.

6. Sung, N. *et al.* Crystal growth and intrinsic magnetic behaviour of Sr_2IrO_4. *Philos. Mag.* **96**, 413–426 (2016). URL http://dx.doi.org/10.1080/14786435.2015.1134835. DOI 10.1080/14786435.2015.1134835.

7. Gretarsson, H. *et al.* Two-magnon raman scattering and pseudospin-lattice interactions in Sr_2IrO_4 and $Sr_3Ir_2O_7$. *Phys. Rev. Lett.* **116**, 136401 (2016). URL http://link.aps.org/doi/10.1103/PhysRevLett.116.136401. DOI 10.1103/PhysRevLett.116.136401.
